# A school based study of psychological disturbance in children following the Omagh bomb

**DOI:** 10.1186/1753-2000-7-36

**Published:** 2013-10-27

**Authors:** Maura McDermott, Michael Duffy, Andy Percy, Michael Fitzgerald, Claire Cole

**Affiliations:** 1 Western Health and Social Services Trust, Omagh, Northern Ireland; 2 School of Sociology Social Policy & Social Work, 6 College Park, Queens University Belfast, Belfast, BT7 1LP, Northern Ireland; 3 Institute of Child Care Research, Queens University Belfast, Belfast, Northern Ireland; 4 Trinity College Dublin, Dublin, Ireland

**Keywords:** Children, PTSD, Bombing

## Abstract

**Objective:**

To assess the extent and nature of psychiatric morbidity among children (aged 8 to 13 years) 15 months after a car bomb explosion in the town of Omagh, Northern Ireland.

**Method:**

A survey was conducted of 1945 school children attending 13 schools in the Omagh district. Questionnaires included demographic details, measures of exposure, the Horowitz Impact of Events Scale, the Birleson Self-Rating Depression Scale, and the Spence Children’s Anxiety Scale.

**Results:**

Children directly exposed to the bomb reported higher levels of probable PTSD (70%), and psychological distress than those not exposed. Direct exposure was more closely associated with an increase in PTSD symptoms than in general psychiatric distress. Significant predictors of increased IES scores included being male, witnessing people injured and reporting a perceived life threat but when co-morbid anxiety and depression are included as potential predictors anxiety remains the only significant predictor of PTSD scores.

**Conclusions:**

School-based studies are a potentially valuable means of screening and assessing for PTSD in children after large-scale tragedies. Assessment should consider type of exposure, perceived life threat and other co-morbid anxiety as risk factors for PTSD.

## Background

Children experience a range of psychological reactions to traumatic events including anxiety, depression and behaviour problems. It is now recognised that the broad categories of PTSD symptoms (re-experiencing, avoidance/numbing and increased arousal) are present in children as well as in adults [1]. In children from the age of 8–10 years post traumatic reactions are similar to those of adults [2] although the DSM diagnostic criteria descriptors are more age appropriate [3]. The reactions in children below 8 years of age and particularly below the age of 5 years to traumatic events are less clear [4]. The purpose of this study was to consider the emotional reactions of children from the age of 8–13 fifteen months after the Omagh bomb.

### The Omagh bombing

On 15 August 1998, the largest single atrocity of the Northern Ireland conflict took place in Omagh, a market town with a population of 26,000, when a car bomb exploded in the town centre. Thirty-one people, including two unborn children (twins) were killed, 382 people were injured of which 135 were hospitalised. Twenty-six families were bereaved. Of those killed, 15 were aged 17 years or under. The bomb had a devastating effect on the community. A large number of those killed or injured were children and young people or adults with young families. Many children and young people sustained injuries resulting in the loss of limbs, loss of soft tissue, scarring and disfigurement. Many more were exposed to scenes of intense horror and suffering.

The first aim of this study was to assess the extent of psychiatric morbidity among children (aged 8 to 13 years) in a community following a car bomb explosion in the town centre on a busy Saturday afternoon. Children under eight were not included because of the different presentation of trauma reactions in these younger age groups [4]. Children and adolescents over the age of thirteen were included in another study to be reported at a later stage with more age appropriate measures. Secondly, we consider if type of exposure to a traumatic event increases PTSD symptoms in children to a greater extent than symptoms of general emotional distress. Thirdly, we investigate which individual and trauma characteristics identified within this study predict PTSD, depression and anxiety, and consider how our findings compare with the risk factors for PTSD in children and adolescents reported in Trickey and colleagues' recent meta-analysis [5] and other studies.

In relation to the first aim, most epidemiological studies have been of adults and older young people, such as the U.S. National Comorbidity Survey [6] that reported a 10% lifetime prevalence rate. In the U.K. National Mental Health Survey [7] a PTSD rate of 0.4% was found in children aged between 11-15 but scarcely registered below the age of 10 years. However the U.K. study reported a point prevalence estimate and the screening instrument used was not PTSD specific. Fletcher [8] in a meta-analysis of 34 studies reported that 36% of children who had experienced a range of traumas met criteria for PTSD. However, the rates of PTSD associated with traumatic events vary considerably from 0% to 100% [9]. In one review of natural disasters [10] 5-10% of children and adolescents met full criteria for PTSD and after road traffic accidents rates of 25 -30% have been recorded [11].

It has been established in many studies that increased exposure is associated with increased mental health problems including PTSD. In a review of 25 studies Foy and colleagues [12] found exposure to be one of three factors (severity of trauma exposure, trauma-related parental distress, and temporal proximity to trauma) that consistently mediated PTSD development in children. A relationship between level of exposure and PTSD has been found in studies of natural disasters [13-15] community violence [16,17] and political conflict [18-20]. Higher PTSD rates have been reported in relation to specific characteristics of traumatic events, for example rates of 90% have been recorded following exposure to gruesome scenes [21]. In warfare studies of PTSD in children, incidence rates between 25% to 70% are reported depending on type of exposure and type of warfare [2,22]. A number of studies have reported level of exposure and trauma severity as two main risk factors of PTSD [12,23-25]. Trickey and colleagues [5] have identified trauma severity as the trauma characteristic most strongly associated with risk of PTSD in children and adolescents but suggest that trauma severity may be difficult to differentiate from trauma exposure. This poses the possibility of a range of psychological effects associated with a wider range of exposure categories including sub categories of direct exposure based on characteristics like proximity to the potentially traumatic event or being present at the time as opposed to just after an incident. Other established peri traumatic risk factors for PTSD such as physical injury [5], exposure to dead bodies [26] and perceived life threat [5] are theoretically more likely with more "direct" exposure such as being present at the time of a bombing compared with less direct exposure witnessing the immediate aftermath of a bomb. There is also evidence that other forms of indirect exposure such as exposure by media [27,28] are linked to increased risk of PTSD. One concept that previous research does not appear to have systematically addressed is the psychological impact on children who are in the vicinity of an event such as a bomb but narrowly miss being at the precise location during or immediately after the event. We have defined this as a "Near Miss" category for analysis in this paper.

With respect to the third aim of this paper we consider how pre, peri and post trauma factors predict psychological reactions, particularly PTSD, in children following the Omagh bomb. In a recent comprehensive meta-analysis of risk factors for PTSD in children, Trickey and colleague's [5] reported risk factors for PTSD as follows: a small effect size for race and younger age; a small to medium-sized effect for female gender, low intelligence, low SES, pre and post-trauma life events, pre-trauma psychological problems in the individual and parent, pre-trauma low self-esteem, post-trauma parental psychological problems, bereavement, time post-trauma, trauma severity, and exposure to the event by media; and a large effect for low social support, peri trauma fear, perceived life threat, social withdrawal, comorbid psychological problem, poor family functioning, distraction, PTSD at time 1, and thought suppression.

In terms of pre-trauma factors, there have been contradictory findings from studies in relation to age [23,29-31]. Trickey and colleagues [5] reported that younger age is largely unrelated to whether a young person develops PTSD but moderator analysis discovered that there was a statistically significant stronger relationship when the trauma was unintentional although the population effect size remained non-significant regardless of whether the trauma was intentional or non-intentional. Trickey and colleagues [5] also reported that younger age was a significant risk factor, with a small effect, if the index trauma was a group event rather than an individual one. There have also been conflicting findings regarding the relationship between gender and PTSD with some studies recording PTSD in girls at twice the rate as in boys [7]. Whist several studies have reported gender as a significant risk factor [12,21,24,29,32], Trickey and colleagues [5] reported female gender to be a consistent although statistically small risk factor and a stronger risk factor in older children and adolescents and also when the trauma is unintentional. Whilst girls seem more vulnerable to internalizing stress reactions, boys display more externalizing behaviour disturbance [24,33]. Several studies have identified a number of pre-trauma risk factors including; prior traumas [20] prior psychiatric problems [25,32,34] and family cohesion [35]. Whilst type and severity of exposure are recognised as important predictors of PTSD in adults and children, studies have reported other specific peri-trauma factors including: a strong acute trauma response [23,36,37], witnessing dead people [26], being physically injured [10] and perceived life threat [24,36,37]. Post trauma factors associated with PTSD in children include: social support [25] and co-morbidity, especially depression and generalised anxiety [38-40].

## Method

Full ethical approval for the survey was granted by the Sperrin Lakeland Health & Social Care Trust which was the relevant ethical and institutional body at the time (1999). The Trust secured the agreement and assistance of the Western Education & Library Board, the main regulatory body for schools in the Omagh area and school principals to survey children in the classrooms. A passive consent procedure was used to obtain parental consent, that is to say all parents were informed of the study and asked to reply, via prepaid envelope, if they wished their child to be excluded from the study. Parents who consented to their child’s inclusion did not have to reply. The parents of bereaved children, children who were hospitalised or children already receiving therapy were contacted directly by members of the Omagh Trauma and Recovery Team and informed of the study. The Omagh Trauma and Recovery Team received 130 referrals for clients aged under 18 between August 1998 and May 2001 [41].

Data was collected 15 months after the car bomb and involved close collaboration between local education and health authorities. All school children aged between 8 and 13 years who were registered within mainstream primary schools within the Omagh area were eligible for inclusion. Thirteen schools participated in the study, with only one school refusing, providing a response rate in excess of 90 per cent. Data was collected via a self-completion booklet and completed by children in their classrooms within schools. All fieldwork was undertaken and supervised by a professional survey organisation and local child and adolescent mental health professionals were available in each school at the time of completion. Table 1 provides details of the characteristics of the children who participated in the survey (n = 1945). The mean age of respondents was 11, and contains slightly more girls than boys. The majority of children lived with both parents (85.3%) and in family units where both parents were employed (75.1%) (Table 1).

**Table 1:** Sample characteristics

**Characteristic**	**Mean**	**SD**	**Proportion**
Age (Mean)	11.4	1.44	
IES (Mean)	15.65	9.73	
BDS (Mean)	8.67	5.22	
SCAS (Mean)	27.42	17.26	
Gender			
*Male*			48.7
*Female*			51.3
Previous psychological treatment (*yes*)			2.9
Physically injured (*yes*)			1.2
Perceived life threat (*yes*)			1.5
Witnessed serious injury (*yes*)			11.1
Witness people dying (*yes*)			7.6
Witnessed people dead (*yes*)			5.6
Post-event support (*yes*)			2.3
Family structure			
*Living with both parents*			85.3
*Living with single parent*			10.8
*Reconstituted family*			3.1
*In state or foster care*			0.7
Parental employment			
*Both parents employed*			75.1
*Mother employed* - *father unemployed*			1.5
*Father employed* - *mother unemployed*			17.7
*Both parents not employed*			5.8

### Measures

*Exposure to the bomb*: Eight items covered various aspects of exposure to the bombing (see Table 8 in Appendix 1). On the basis of responses to these items, respondents were classified as belonging to one of five mutually exclusive exposure categories. “Exposed - in town at time" means was in Omagh town when the bomb exploded and witnessed injury or death of others or was directly harmed. “Exposed - in town after” means was in Omagh town shortly after the bomb exploded and witnessed injury or death of others or was directly harmed. “Loss” means did not witness injury or death of others, not injured but experienced loss or injury of someone close (family, relative or friend). “Near miss” means was in Omagh town when the bomb exploded but did not witness injury or death of others, was not directly harmed and did not experience loss. “No exposure” means was not in Omagh town when or after the bomb exploded, was not a witness and did not experience loss. In addition, children reported whether they had received any physical injuries (*physically injured*) or thought they were going to die (*perceived life threat*).

*The Impact of Event Scale* (*IES*) [42] is a widely used screening test for PTSD in children. In this study, the 8 item CRIES-8 (which lacks any arousal items) was used (α=0.82) as it was found to be as efficient as the CRIES-13 (which includes arousal items) in classifying children with and without PTSD [43]. It provides a continuous score for overall PSTD, and two sub-scales each consisting of four items: (1) intrusive thoughts, memories and images and (2) avoidance of thoughts and reminders. Items were grounded in the Omagh Bombing and referenced to experiences within the previous seven days.

*The Birleson Depression Self*-*Rating Scale for Children* (*BDS*) [44] is an 18-item scale assessing the level of depression in children (α=0.82). Items were scored on a three point scale (0,1,2). Responses include ‘most’, ‘sometimes’ and ‘never’. A score of 0 indicated a healthy response and a score of 2 indicated an unhealthy or depressed response.

*The Spence Children*’*s Anxiety Scale* (SCAS) [45] consists of 38 items on specific anxiety symptoms with a further six filler items (α=0.94). Responses include ‘never’, ‘sometimes’ , ‘often’ and ‘always’ and are recorded on a four-point scale (0,1,2,3). The scale provides a global anxiety rating together with scores on six individual subscales covering specific anxiety symptoms, namely separation anxiety, social phobia, obsessive-compulsive disorder, panic/agoraphobia, generalised anxiety, and, fears of physical injury.

*Socio*-*demographics*: Each respondent provided details of their age and gender, as well as information on family structure (living with both parents/living with single parent/reconstituted family/in state or foster care) and parental employment (both parents employed/mother employed and father not employed/father employed and mother not employed/both parents not employed) (Table 1). Post event support was measured by asking if help was received because of difficulties experienced following the bomb and a checklist of sources of help was provided to identify the provider(s).

### Statistical analysis

A series of OLS regression models were estimated to examine the predictors of PTSD, anxiety and depression. A three step hierarchical regression was conducted with the predictor variable included in blocks corresponding to pre-, peri- and post-trauma variables. These models were restricted to those individuals who were in town on the day of the bombing and/or witnessed traumatic events. As the sample was clustered at the school level, school dummy variables were included in the model to account for the lack of independence due to school clustering. This ensures that the regression standard errors are adjusted for the lack of independence at the school level. While these dummy variables were included within the model they were not reported within the presented regression tables. None of the school level dummies were significant within the various models.

## Results

### Psychiatric morbidity

Forty seven per cent of the sample met probable clinical PTSD caseness according to IES scores. Using a BDS score of 18 or above, 6% of children in the study met clinical caseness for probable depression and using a cut off score of 60 or more on the SCAS responses 5.7% of the children met clinical caseness for probable anxiety (Table 2).

**Table 2:** **Probable caseness rates for PTSD** (**IES**), **depression** (**BDS**) **and anxiety** (**SCAS**)

**Type of exposure**	**IES**			**BDS**			**SCAS**		
	**Low**	**High**	**%**	**Low**	**High**	**%**	**Low**	**High**	**%**
No exposure	603	330	35.4	862	41	4.5	896	37	4.0
Near miss	10	10	50.0	19	1	5.0	20	0	0.0
Loss	353	404	53.4	683	46	6.3	716	42	5.5
Exposed - in town after	43	87	66.9	111	12	9.8	118	12	9.2
Exposed - in town at time	26	75	74.3	86	11	11.3	87	14	13.9
	r=113.911, p<0.001			r=11.664, p<0.05			r=22.791, p<0.001		

### Type of exposure: associations with PTSD and other psychiatric disorders

Over half the children surveyed had some form of exposure to the bombing (52%) (Table 3). This was mainly in the form of loss of a family member, relative or friend (39%), however, over one in ten children did witness the aftermath of the bomb blast. Around one per cent of children were directly injured in the blast, with two per cent thinking they were actually going to die (Table 1).

**Table 3:** Type of exposure experienced by participants

**Type of exposure**	%
No exposure	48.1
Near miss	1.0
Loss	39.0
Exposed - in town after	6.7
Exposed - in town at time	5.2

**Figure 1:**
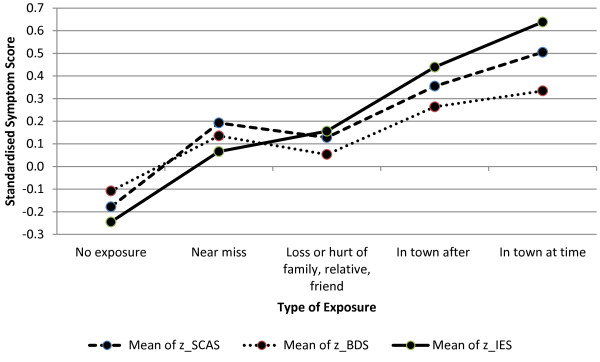
**SCAS BDS and IES Standard Scores for types of exposure.** A graphical representation of the Impact of Events (IES), Spence Children's Anxiety Scale (SCAS) and Birleson Depression Self Rating Scale for Children standardized symptom scores for each type of exposure to the Omagh Bomb.

No age or gender variations were noted across the levels of exposure (Table 4). The mean scores on the IES, BDS and the SCAS were 15.65, 8.67 and 27.42 respectively (Table 1). The PTSD, depression and anxiety scores varied significantly across types of exposure, with increased exposure associated with higher scores on the IES, BDS and SCAS (Table 4). There were significant differences between the level of exposure and PTSD symptoms (F(4,1856)=37.698, p<0.01), depression (F(4,1867)=8.138, p<0.01) and anxiety (F(4,1778)=18.179, p<0.01). Figure 1 shows the IES, SCAS and BDS standardised symptom scores for each type of exposure. An increase in level of exposure is associated with increased levels of PTSD. However, those in the near miss group exhibited higher levels of anxiety and depression than the loss group. Direct exposure (those present at the time of the explosion and those present after the explosion) was associated with larger increases for PTSD symptoms than for general psychiatric distress. Paired comparisons of these differences showed that standardised IES scores of the two groups directly exposed differed significantly compared to the loss group (p<0.01), no exposure (p<0.01) and the near miss group (p<0.05). The differences between the two groups directly exposed to the bomb scenes, (those present at the time of explosion and those present after the explosion) were not significant on the IES (p=0.255), SCAS (p=0.663) and depression measures (p=0.604). The anxiety scores (SCAS) of those in the two exposure groups were significantly different to those in the no exposure (p<0.01), loss (p<0.01) groups but not the near miss (p=0.334) group. On the depression measure (BDS) those directly exposed differed significantly compared to the loss group (p<0.01) and the no exposure group (p<0.01), but not the near miss group (p=0.494).

**Table 4:** Sample characteristics by exposure to the bombing

	**Type of exposure**					**p**
**Characteristic**	**No exposure**	**Near miss**	**Loss**	**Exposed** - **in town after**	**Exposed** - **in town at time**	
Age (Mean)	11.5	11.3	11.4	11.3	11.2	0.233
IES (Mean)	13.17	16.05	17.07	20.29	21.73	0.00**
BDS (Mean)	8.08	9.35	8.92	10.02	10.38	0.00**
SCAS (Mean)	24.23	30.37	29.19	33.85	34.89	0.00**
Female (%)	50.7	55.0	51.3	53.8	52.5	0.959
Previous psychological treatment (*yes*)	1.3	0.0	3.6	6.9	8.2	0.00**
Post-event support (*yes*)	0.7	0.0	1.9	7.0	15.0	0.00**
Perceived life threat (*yes*)	-	0.0	-	6.0	21.1	0.003**
Physically injured (*yes*)	-		-	10.9	9.9	
Witnessed serious injury (*yes*)	-		-	92.1	98.0	
Witness people dying (*yes*)	-		-	61.1	70.3	
Witnessed people dead (*yes*)	-		-	39.8	57.4	

Note: “Exposed - in town at time" means in Omagh town when the bomb exploded and witnessed injury or death of others or was directly harmed. “Exposed - in town after” means in Omagh town shortly after the bomb exploded and witnessed injury or death of others or was directly harmed. “Near miss” means in Omagh town when the bomb exploded but did not witness injury or death of others and was not directly harmed. “Loss” means experienced loss or injury of someone close (family, relative or friend) but no direct harm. “No exposure” means not in Omagh town at the time or shortly after the bomb exploded, not a witness and did not experience loss. “IES”: Impact of Events Scale; “BDS”: Birleson Depression Scale: “SCAS”: Spence Children’s Anxiety Scale. P: *significant at the 0.05 level; **significant at the 0.01 level.

### Predictors of PTSD and other psychiatric disorders

Significant predictors of increased IES scores included being male, witnessing people injured and reporting a perceived life threat (Table 5; model 2). However, when co-morbid anxiety and depression are included as potential predictors (see Table 5; model 3), gender, exposure to injury and life threat no longer remain significant predictors. In model 3, anxiety remains the only significant predictor of PTSD scores.

**Table 5:** **Predictors of PTSD symptoms 15 months after explosion among children present in Omagh** (**N**= **212**)

	**Model 1**		**Model 2**		**Model 3**	
**Variable**	**β**	**p**	**β**	**p**	**β**	**p**
Pre-trauma						
Age	0.229	0.692	0.190	0.734	0.554	0.285
Male	−4.172	0.011*	−3.297	0.036*	−0.495	0.741
Previous psychological treatment	3.473	0.171	3.543	0.148	1.472	0.518
Peri-trauma						
Perceived life threat			5.692	0.015*	2.541	0.250
Physically injured			−1.153	0.679	−0.331	0.899
Witnessed serious injury			4.077	0.015*	2.519	0.103
Witness people dying			0.952	0.589	1.712	0.295
Witnessed people dead			1.764	0.315	0.650	0.688
Post-trauma						
Post-event support					−1.710	0.506
Depression (BDS score)					0.152	0.270
Anxiety (SCAS score)					0.173	0.000**

Notes:

1. PTSD symptoms measured by Impact of Events Scale (IES) score.

2. Those present in Omagh includes those in Omagh town centre when the bomb exploded or shortly after and/or witnessed related traumatic events.

3. Dummy variables for school included in model but excluded from the table.

4. Model 1 Adjusted R^2^ =0.004; Model 2 Adjusted R^2^ = 0.107; Model 3 Adjusted R^2^ = 0.252.

5. P: *significant at the 0.05 level; **significant at the 0.01 level.

Age and gender were significant predictors of probable anxiety, with younger children and girls reporting significantly higher anxiety scores (Table 6). Perceived life threat, witnessing injuries and receiving post bombing psychological support were also significantly associated with higher levels of overall anxiety.

**Table 6:** **Predictors of anxiety 15 months after explosion among children present in Omagh** (**N**= **222**)

	**Model 1**		**Model 2**		**Model 3**	
**Variable**	**β**	**p**	**β**	**p**	**β**	**p**
Pre-trauma						
Age	−2.244	0.059	−2.396	0.039*	−2.331	0.041*
Male	−15.313	0.000**	−14.067	0.000**	−14.271	0.000**
Previous psychological treatment	10.317	0.052	10.607	0.039*	8.604	0.091
Peri-trauma						
Perceived life threat			15.467	0.001**	13.151	0.006**
Physically injured			−3.812	0.515	−6.784	0.246
Witnessed serious injury			7.621	0.028*	7.244	0.034*
Witness people dying			−2.235	0.531	−3.271	0.354
Witnessed people dead			5.334	0.133	4.849	0.165
Post-trauma						
Post-event support					16.651	0.004**

Notes:

1. Anxiety symptoms measured by Spence Childhood Anxiety Scale (SCAS) score.

2. Those present in includes those in Omagh town centre when the bomb exploded or shortly after and/or witnessed related traumatic events.

3. Dummy variables for school included in model but excluded from the table.

4. Model 1 Adjusted R^2^ =0.096; Model 2 Adjusted R^2^ = 0.180; Model 3 Adjusted R^2^ = 0.210.

5. P: *significant at the 0.05 level; **significant at the 0.01 level.

Being female was also a significant predictor of higher depression score, as was witnessing injury (Table 7). However, even after controlling for witnessing injury and death, the experience of witnessing people you thought were dying was associated with lower depression scores.

**Table 7:** **Predictors of depression 15 months after explosion among children present in Omagh** (**N**= **241**)

	**Model 1**		**Model 2**		**Model 3**	
**Variable**	**β**	**p**	**β**	**p**	**β**	**p**
Pre-trauma						
Age	−0.036	0.903	-.005	0.987	0.015	0.960
Male	−2.640	0.003**	−2.338	0.007**	−2.418	0.005**
Previous psychological treatment	2.488	0.066	2.308	0.083	1.932	0.152
Peri-trauma						
Perceived life threat			2.581	0.037*	2.225	0.077
Physically injured			1.516	0.311	1.040	0.495
Witnessed serious injury			1.842	0.042*	1.884	0.037*
Witness people dying			−2.151	0.019*	−2.348	0.011*
Witnessed people dead			1.591	0.085	1.544	0.093
Post-trauma						
Post-event support					2.084	0.135

Notes:

1. Anxiety symptoms measured by Birleson Depression Scale (BDS) score.

2. Those present in Omagh includes those in Omagh town centre when the bomb exploded or shortly after and/or witnessed related traumatic events.

3. Dummy variables for school included in model but excluded from the table.

4. Model 1 Adjusted R^2^ =0.032; Model 2 Adjusted R^2^ = 0.081; Model 3 Adjusted R^2^ = 0.086.

5. P: *significant at the 0.05 level; **significant at the 0.01 level.

Of those directly exposed to the bomb approximately one in ten received post-event psychological/psychiatric interventions. Post-event support significantly predicted probable anxiety (p<0.01; Table 6) but not PTSD (Table 5) or depression (Table 7). Those who received support had significantly higher levels of depression (t(216)=3.007, p<0.01) and anxiety (t(201)=3.656, p<0.01). However, no significant differences in PTSD scores were observed (p=0.057).

## Discussion

The first aim of the present study was to assess the extent and nature of psychiatric morbidity among children (aged 8 to 13 years) 15 months after a car bomb explosion. The results suggest high levels of psychiatric morbidity, particularly probable PTSD, in the children. Even with the general reduction in the levels of PTSD reactions that tends to occur with time [20,35] and the relatively low numbers with direct exposure, the levels of probable PTSD reported in this study would appear to be high [6-8] and in line with rates found in warfare studies of children [2,22]. A number of factors may be relevant to this finding. First, the location of the incident was outside shops in the main street in the centre of a small market town and many school children will have continued to pass by the bombsite on a regular basis, providing a continual reminder of the incident and recurrent trigger of trauma memories. Secondly, the bombing was unexpected in the context of the ongoing political process, coming just four months after an agreement was signed between the British and Irish Governments that provided a basis for a political settlement and reform. In the preceding months the main paramilitary groups had declared ceasefires raising hopes and expectations that a period of peace had begun. After the explosion many children and young people reported that they thought the bomb alert was merely a hoax. Furthermore, telephone warnings of the explosion were provided which was an established practice during the Northern Ireland conflict to ensure the area under threat is evacuated. However on this occasion, ambiguous information about the location of the bomb misled the police who unintentionally moved some people towards the car containing the explosive device. After the incident, it was frequently reported that the sense of shock was intense because people believed they were standing in a safe place, not beside the car that contained the bomb. Many children and families were moved to streets nearby and were not directly exposed to the explosion but a theme that dominated the media reports afterwards was how many more might easily have been unintentionally diverted to stand beside the car bomb. In the days that followed the explosion these items about intentionality which has been linked with PTSD in younger age [21] and confusion about the location of the bomb were repeatedly discussed in the media and throughout the Omagh community.

Also, the group nature of the Omagh bombing may have contributed to higher rates of probable PTSD, which is consistent Trickey and colleague's meta-analysis [5] that found group trauma to be significant for younger children compared to individual trauma. It is also possible that a number of the children were subsequently re-exposed to distress in the days and months following the bomb in the 15 months prior to the data being collected. In addition to potential stressors linked to more normal life events, during the weeks that followed the Omagh bombing a repeated series of hoax phone calls to the local police led to the town centre being evacuated on a number of occasions. Some studies suggest that young people are vulnerable to relapse if exposed to such subsequent stressors [20,35].

Our second aim was to consider if type of exposure to a traumatic event increases PTSD symptoms in children to a greater extent than symptoms of general emotional distress. Our findings that children exposed to the bomb reported higher levels of probable PTSD and psychological distress than those not exposed (Figure 1) supports the findings from other studies [24,25]. Our study also indicates that direct exposure is more closely associated with an increase in PTSD symptoms than general psychiatric distress (Figure 1). Our finding that there is a trend, albeit non-significant, for an increase in PTSD and general psychiatric distress with increased exposure type (higher rates for "being present at the time" as opposed to "being present after" the explosion) provides some support for the finding from Foy and colleague's review [12] that temporal proximity is an important mediator of PTSD in children.

A novel consideration in our study is the concept of near miss which as far as we can discover has not been extensively researched in children. In this study the data suggests that the near miss group (those children who were in town but missed the explosion and the aftermath) differed significantly on the PTSD measure from those children directly exposed (p<0.05) but did not differ significantly on the PTSD measure from the loss group (p=0.630) or the no exposure group (p=0.174). Children in the near miss group, however, did not differ significantly from the direct exposure groups in their depression symptom levels (p= 0.432) or anxiety symptom levels (p=0.334), whereas those in the loss and no exposure groups had significantly lower levels of general psychiatric distress compared with those directly exposed. The mean IES score is higher in the loss group than the near miss group but the differences on all measures between the near miss and loss group were not statistically significant. However we have to be cautious about these findings because of the small number in the “near miss” category (N= 20) and the restricted statistical power to calculate differences with this group. These "near miss" findings are similar to the findings of a community study of adults after the Omagh bombing [46] which found that those in the "near miss" group did not differ in PTSD or general psychiatric measures from those who had no exposure.

Our third aim was to consider which individual and trauma characteristics predict chronic PTSD symptoms. In relation to pre-trauma factors, our finding that age was a predictor of probable anxiety but not a predictor specifically of probable PTSD supports the findings from a number of previous studies [29,31,35] but we accept that the age range in our analysis was restricted to children and did not include adolescents. Only a small effect was reported for younger age by Trickey and colleague's [5] and our finding supports their conclusion that younger age is largely unrelated to whether a young person develops PTSD. Female gender has been reported as a small but significant risk factor for PTSD in adults [47] and children [5]. However, in our study when co-morbidity and post trauma support are controlled for in the analysis, the association between gender and PTSD is no longer significant. As discussed earlier, Trickey and colleagues [5] reported that younger age has a moderating effect on gender as a risk factor for PTSD in children. In this study girls reported higher levels of probable depression and anxiety than boys and these associations remained significant after peri- and post trauma factors were added to the regression analysis (Models 2 and 3, Tables 6 and 7). Similar gender differences were reported in another study of school children in Belfast after a bomb had destroyed their school [48] as indicated earlier, recognised that negative affect is often externalised in boys in the form of behavioural symptoms [32].

Peri traumatic factors that significantly predicted increased IES scores in this study were witnessing people injured and reporting a perceived life threat. However, when co-morbidity and post trauma support were controlled for, these peri-traumatic factors were no longer significant. Children who witnessed injured people were also at higher risk of depression. These findings are consistent with other studies [29] and both factors were reported as risk factors with large effect sizes in Trickey and colleagues' meta-analysis [5]. Of those children who witnessed the aftermath of the bomb, almost all saw people injured, almost half those exposed saw people they thought were dead and one in ten received psychological/psychiatric interventions post-event. This exposure to such gruesome scenes may contribute to the high rates of probable PTSD for the exposure groups as found in other conflict related studies where PTSD rates as high as 87% [30] and 90% [21] were reported. However, it is interesting that the only significant exposure predictor in our study was "seeing people injured" which was a significant predictor on all 3 outcome measures the IES, SCAS and BDS. In the Omagh bomb a large number of children and young people suffered burns and shrapnel injuries resulting for some in permanent disabilities including loss of sight and amputated limbs.

Post-trauma factors that were considered included “support received for difficulties experienced following the bomb” which was significantly associated with anxiety but not specifically PTSD or depression. Those receiving post-event interventions who were present in Omagh and exposed to the bomb had significantly higher depression and anxiety scores compared with those not receiving post-event support, however, no differences in PTSD scores were noted. Social support has been reported elsewhere as a risk factor for PTSD with a large effect size in both adults [47] and children [5]. Our finding is interesting because the Omagh bombing occurred in a changed political context, an early phase of peace-building with the main paramilitary groups on ceasefire, and so the social policy response was different to previous events. In the aftermath of the tragedy, political leaders and many celebrities visited the town and thousands of people attended vigils and memorial services. Government funding was made available specifically to provide supports for the bomb victims and to co-ordinate a response involving health, social, educational agencies and voluntary, faith and community groups. Despite these policy and community initiatives, whilst our study found that social support was linked to anxiety this factor did not appear to have had an effect specifically on traumatic symptoms in younger children.

Co-morbid psychological problems have been reported as risk factors with large effects in Trickey and colleagues' meta analysis [5]. In our study, of those children classified as reaching PTSD caseness, 10% also met probable caseness for anxiety and 9% probable caseness for depression. Over one third (38%) of those children reaching probable depression caseness also met probable caseness for anxiety. Co-morbid psychological problems had a moderating effect on pre-trauma characteristics and exposure factors in predicting probable PTSD. Our findings are consistent with other studies that have identified co-morbid symptoms as amongst the highest risk factors for chronic post trauma distress in children [49].

## Conclusions

High rates of PTSD have been found in studies of children living in conflict areas [19,30,35]. Similar to patterns in adults [6] chronic post-trauma symptoms persist in a substantial sub-group of children and can severely interfere with functioning [20,50,51]. It is important that these children, whose needs may not be fully recognised and under-reported by parents [2], are identified as early as possible and offered effective therapies and support. Our study is one of a growing number of school-based studies that have been organised after single incident traumas for screening and assessing children [16,20] and providing early treatment responses [52]. Our findings that witnessing people injured and reporting a perceived life threat were significant risk factors and that co-morbid anxiety mediates the effect of exposure, age and gender as predictors of PTSD adds to the growing literature base identifying specific key factors for screening and assessing children after traumatic events.

### Limitations

Our data was gathered 15 months after the bomb so it is likely that screening in the immediate aftermath of the bomb would have identified higher levels of PTSD symptomotology. Our questionnaire did not capture any traumas or significant life events that children may have been experienced in the intervening period that may have compounded an initial traumatic reaction to the bombing. Self-report questionnaires were used in the screening and we recognise these are only an indicator of probable psychiatric disorders and do not provide a complete accurate diagnosis. We were unable to collect multi-informant data from parents or teachers which would have provided confirmatory data to identify morbidity amongst the sample. While the overall sample size was large, the number of children who were directly exposed to the bombing was relatively small. This will have reduced the statistical power of the regression models. Finally, the study assessed psychological symptoms but did not measure the impact of symptoms on daily functioning.

## Appendix 1

Table 8 illustrates how the responses on the questionnaire were classified into exposure categories

**Table 8:** Items included in development of exposure measure

**Item**	**No exposure**^**1**^	**Near miss**	**Loss**^**2,**^^**3**^	**Exposed - in town after**^**4**^	**Exposed – in town at time**^**5**^
**Proximity**					
Were you in Omagh town centre when the bomb exploded?	✗	✓	-	✗	✓
Were you in Omagh town centre shortly after the bomb exploded?	✗	-	-	✓	-
**Injured**					
Were you hurt?		✗	✗	-	-
**Loss**					
Did any of your family, relatives or friends die?	✗	✗	✓	-	-
Did you know anyone else who died?	✗	✗	✓	-	-
**Witness**					
Did you see people you thought were seriously hurt?		✗	✗	-	-
Did you see people you thought were going to die?		✗	✗	-	-
Did you see people you thought were dead?		✗	✗	-	-

✓ Positive response required for inclusion in category.

✗ Negative response required for inclusion in category.

- Either positive or negative response valid.

^1^Items with blank cells were filtered out for children who were not in Omagh on the day of the bomb.

^2^A positive response to at least one of the two questions relating to loss was required for inclusion in this category.

^3^Children who experienced loss but were in town on the day of the bomb and witnessed traumatic events or were injured were included in one of the exposure categories.

^4^A positive response to at least one of the witness questions or the injured question was required for inclusion in this category.

^5^A positive response to at least one of the witness questions or the injured question was required for inclusion in this category.

## Competing interests

The authors declare they have no competing interests.

## Authors’ contributions

MMcD, MD, AP and MF designed the study, MMcD, AP and MD collected the data, MD, AP and CC analysed the data and drafted the paper. All authors contributed to writing, and read and approved the final manuscript.
